# The effect of dietary patterns on maternal anaemia in North Shewa, Ethiopia: A case–control study with Propensity Score Analysis

**DOI:** 10.1177/02601060231152345

**Published:** 2023-01-23

**Authors:** Kelemu Tilahun Kibret, Catherine Chojenta, Ellie D’Arcy, Deborah Loxton

**Affiliations:** 1 Priority Research Centre for Generational Health and Aging, School of Medicine and Public Health, University of Newcastle, Australia; 2 Integrated Primary Care and Partnerships, Western NSW Local Health District, Australia

**Keywords:** Anaemia, dietary patterns, dietary diversity, pregnant women, case–control study

## Abstract

**Objective:**

This study aimed to assess the effect of dietary patterns during pregnancy on anaemia.

**Design, Setting and Participants:**

A case–control study with propensity score analysis was conducted among pregnant women selected from five health facilities in North Shewa Zone, Ethiopia from November 2018 to March 2019. A multivariable conditional logistic regression model was applied after propensity score matching to assess the effect of dietary patterns on anaemia, and a *p* < 0.05 was taken as significant. Four hundred and seventeen pregnant women were included (105 cases and 312 controls) with a 1:3 case-to-control ratio. Cases were pregnant women with a haemoglobin level <11 gram/Deci litter (g/dL), and controls were pregnant women with a haemoglobin level ≥11.0 g/dL.

**Results:**

A low dietary diversity score (adjusted odd ratio (AOR) = 2.14; 95% confidence interval (CI): 1.24, 3.69), reducing food intake (AOR = 6.89; 95% CI: 3.23, 14.70) and having no formal education (AOR = 3.13; 95% CI: 1.18, 8.32) were associated with higher odds of anaemia among pregnant women.

**Conclusions:**

During pregnancy, intake of a low diversified diet, reduced food intake and low educational status were associated with higher odds of anaemia. Dietary counselling should be emphasised and strengthened in the existing prenatal health service program, with women strongly encouraged to increase their diversified food intake instead of reducing it during pregnancy.

## Background

Anaemia in pregnant women is defined as a haemoglobin (Hgb) level <11 g/dL at sea level (WHO, 2011). Pregnant mothers are at higher risk of anaemia due to several body changes during pregnancy, such as increased blood volume (Soma-Pillay et al., 2016) with a low increment of red blood cell mass. Thus, a sufficient amount of iron, folate and vitamin B12 are required to make Hgb. Iron absorption in pregnancy is difficult which makes it hard to make Hgb, which then could lead to anaemia (Soma-Pillay et al., 2016).

Anaemia is a major public health problem for pregnant mothers globally (Stevens et al., 2013), with greater rates in low and middle-income countries (LMIC) (Say et al., 2014). Worldwide, in 2011, approximately 38% of pregnant women and 43% of children aged five years and under were classified as anaemic. The highest prevalence rates in LMICs are reported in Central and West Africa (56%), East Africa (36%) (Stevens et al., 2013) and South and South-East Asia (52.5%) (Sunuwar et al., 2020). Similarly, a recent systematic review of 26 articles from developing countries found that around 37%–48% of pregnant women had experienced anaemia (Rahman et al., 2016).

Anaemia is a common problem in Ethiopia: the current Ethiopian Demographic and Health Survey (EDHS 2016) reported a 29% prevalence rate of anaemia among pregnant women (Central Statistical Agency (CSA) [Ethiopia] and ICF, 2016). Similarly, several small-scale studies from different parts of the country reported that prevalence rates of anaemia among pregnant women ranged from as low as 17% in the north (Melku et al., 2014), 32% in the south (Gebremedhin et al., 2014) and up to 44% (Kedir et al., 2013) and 57% (Addis Alene and Mohamed Dohe, 2014) in the east.

Dietary intake is considered to be a modifiable risk factor that may influence the occurrence of anaemia and is one potential area to target for the prevention of anaemia during pregnancy. Previous research has shown that dietary patterns during pregnancy have an effect on anaemia in resource-limited settings (Kedir et al., 2013, Gebremedhin et al., 2014, Alem et al., 2013, Lebso et al., 2017). However, inconsistent results have been reported in other studies (Saaka et al., 2017, Ali et al., 2014), and most of the studies were cross-sectional studies and could not control for all possible confounder factors such as sanitation, water source and gravidity. Moreover, the association between dietary intake and anaemia may be context-dependent and, hence, well-designed research is necessary for this area to properly assess the effect of dietary patterns on maternal anaemia. Understanding the effects of dietary patterns on maternal anaemia may help to tailor local strategies and dietary recommendations for the prevention of maternal anaemia. Thus, this study aims to assess the effects of dietary patterns on maternal anaemia in North Shewa, Ethiopia.

## Methods

This study was conducted and reported following the Strengthening the Reporting of Observational Studies in Epidemiology (STROBE) guidelines for reporting observational studies. The STROBE statement checklist is provided as supplementary material.

### Study settings and periods

This study was conducted across five health facilities in North Shewa, Ethiopia, from November 2018 to March 2019. North Shewa Zone is one of the administrative areas of Amhara Regional State. According to the 2007 census, this zone has a population of 1,837,490, of whom 51% (*n* = 928,694) are men and 49% (*n* = 908,796) are women. It has an area of 15,936.13 square kilometres, and around 12% of the population are urban inhabitants. In this zone, the Amhara are the largest ethnic group (96%); 97% of people speak Amharic as a first language and 95% of the population are Ethiopian Orthodox Christians (Central Statistical Agency, 2012). This zone is subdivided into districts and each district has more than 10 *kebele*s (the lowest administrative unit). The zone has six district primary hospitals, one zonal referral hospital and 95 health centres. In each district, there is at least one health centre, each of which is expected to provide services to more than 25,000 people.

### Study design

A case–control study was used to assess the effect of dietary patterns on maternal anaemia. Cases were all pregnant women with a Hgb level < 11 g/dL and controls were women with a Hgb level ≥ 11.0 g/dL. For each identified case, three healthy controls were selected from each health facility. Participants were recruited consecutively for approximately four months during their antenatal care (ANC) visits from each selected health facility.

### Source and study population

The study population included all pregnant women who had attended ANC follow-up in one of the five selected health facilities and had provided informed consent during the study period (November 2018 to March 2019). Women who were unable to hear or speak, severely ill with medical problems such as cardiovascular or renal diseases or unwilling to participate were not included in the study.

### Sampling method and sample size determination

Five health facilities were selected for this study. The sample size was allocated to each health facility proportional to the average number of ANC visits for the preceding three months (August–October 2018). The study participants were recruited consecutively from each health facility until the allotted sample size was achieved.

The sample size was calculated using different factors of anaemia (intestinal parasite, no education, iron supplementation) and selecting those that produced a maximum sample. To determine the optimum sample size, studies were selected taking into consideration the following points: recently conducted studies, comparable population and study setting, and well-conducted studies (adequate sample size with low non-responses rates and appropriate statistical analysis with control for confounders). The sample size was then calculated using OpenEpi version 3.01 software for unmatched cases control study (openepi.com) using the following parameters: intestinal parasite as the exposure variable, 5% significance level, a power level of 80%, a 1:3 ratio of cases to controls with odd ratio = 1.9 and using double proportion formula (Fleiss et al., 2003). We used a 1:3 case-to-control ratio to increase the statistical power to detect a difference. Therefore, using a 5% contingency level, the final sample size was 417 (105 cases and 312 controls).

### Data collection procedures and measurements

The data were collected using an interviewer-administered questionnaire, anthropometry measurements and a record review of laboratory measurements. The data collectors interviewed the study participants after they had finished the services as a client-exit interview. The questionnaire was adapted from the Food and Agriculture Organization (WHO/FAO, 2001) and EDHS 2016 surveys (Kennedy et al., 2011, Central Statistical Agency (CSA) [Ethiopia] and ICF, 2016) and by reviewing other relevant literature (Asayehu et al., 2016, Kuche et al., 2015). The questionnaire comprised five sections. The first section assessed the respondents’ sociodemographic characteristics: age, religion, occupation, ethnicity, educational status, marital status, place of residence, family size, income, main water source and toilet facilities available in their household. The second section evaluated maternal characteristics, including gravidity/parity, birth interval, stillbirths, ANC visit, nausea/vomiting and alcohol intake. The third section addressed maternal feeding habits/practices, including varying food intake, avoidance of foods, pica practices (ingestion of non-nutritive substances), meal frequency, eating patterns and use of supplements including iron/folate or consumption of de-worming tablets. The fourth section of the questionnaire was designed to examine the dietary intake of study participants. The dietary intake of participants was assessed through the administration of a 24-h recall food questionnaire, adapted from the FAO and 2016 EDHS. Participants were requested to recall all the foods they had consumed in the preceding 24 h. This was done initially and spontaneously by the participant and then through probing questions from the interviewer regarding all foods consumed. The final section of the questionnaire included measurements of maternal anthropometry (height, mid-upper arm circumference (MUAC), weight and gestational age) and a record review of laboratory measurements (Hgb level and stool examination).

### Anthropometric and laboratory measurements

The Hgb levels of participants, which were routinely measured during ANC follow-ups to determine the anaemia status of pregnant women, were taken from ANC registries. A Hgb level below 11 g/dL was classified as anaemia (WHO, 2011). The MUAC was measured by using non-stretchable measuring tapes: the measurement was taken from the relaxed left arm and rounded to the nearest 0.1 cm. The MUAC reflects the past and current nutritional status of a pregnant woman. MUAC cut-off values below 23 cm and greater than or equal to 23 cm were used to classify women as ‘wasting’ or ‘normal’, respectively (WHO, 1995). Women were instructed to wear light clothing and were weighed without shoes (to the nearest 100 g) using a mechanical weight scale. The heights of each study participant were also measured with shoes off. Midwives estimated the gestational age of each participant using the last menstrual period method at the ANC visit to the health facility.

The questionnaire was prepared in English and translated into the Amharic language (a local language in the study area). The training was provided to all data collectors, and the overall activity of the study was closely monitored at each health facility. Data quality was assured through continuous supervision and by using the data collection tools adapted from validated measures/sources.

## Data processing and analysis

The completeness and consistency of the questionnaire were checked in the field and during data entry. Then, the data was cleaned, coded and entered into SPSS version 24. The analysis was conducted using Stata version 14 (StataCorp. 2015, 2015). The dietary diversity score (DDS) was computed using data from 24-h dietary recall, based on the recommendations of the Food and Nutrition Technical Assistance Project (FAO; FHI 360, 2016). Food items and liquids reported in the 24-h dietary recall were categorised into 10 food groups. These food groups were the following: 1) Starchy staples (grains, white roots, tubers); 2) Legumes/pulses (beans, peas and lentils); 3) Nuts and seeds; 4) Dairy; 5) Meat, poultry and fish; 6) Eggs; 7) Dark green leafy vegetables; 8) Vitamin A-rich fruits and vegetables; 9) Other vegetables (tomatoes, onions); and 10) Other fruits. Women who ate a single food item from any of these food groups earned 1 point. If the participant did not consume a food item within the food group, they would receive 0 points for that category. The minimum and maximum DDSs were 0 and 10 points, respectively (FAO; FHI 360, 2016).

## Propensity score analysis

Propensity score analysis was performed to adjust for significant differences in baseline covariates and to have unbiased estimates. The propensity score (Rosenbaum and Rubin, 1985, Rubin, 1983) is the conditional probability of assigning a variable to a particular group (exposed or unexposed) given a set of observed covariates. A propensity score is an essential tool for causal inference in non-experimental studies in which randomisation is impossible and symmetry of treatment/exposure groups is unlikely. Propensity score analysis avoids selection or confounder bias (Stuart, 2010). The propensity score analysis was performed as follows. First, a propensity score for each patient was estimated using the logistic regression model, with dietary diversity during pregnancy as the endpoint (high coded as 1; low, as 0). The following variables were used to estimate the propensity score: age, residence, educational status, marital status, occupation, first pregnancy, ANC, nausea and vomiting, gestational age, eating before pregnancy, change in food after pregnancy, food avoided, started new food types, meal patterns, fasting and craving. The propensity score model was assessed by using the *c* statistic (Westreich et al., 2011) and Hosmer–Lemeshow statistics (Hosmer et al., 2013).

Second, the study subjects were grouped into 10 strata based on the quantiles of population propensity scores (Austin, 2011). In propensity score analysis, the sub-classification method uses all individuals in the dataset, and it is recommended for settings where the outcome data are already available (Stuart, 2010). Third, the main concern with propensity score analysis is the balance of the covariates in the resulting matched data (Stuart, 2010). Thus in this study, the covariate balance between two groups (women with low DDS and women with high DDS) was checked using standardised mean difference (standardised bias) with the ‘pbalchk’ command in Stata (Lunt, 2014). It is recommended that the absolute standardised differences of means should be less than 0.25 (Rubin, 2001).

Finally, using the propensity score-matched data, a conditional logistic regression model was used to assess the association between maternal DDS and anaemia, with adjustment for covariates (Stuart and Green, 2008) to control for the remaining minor differences in the matched sample (Austin, 2009).

Bivariate analysis was undertaken to determine the effect of dietary intake and other factors on anaemia (age, occupation, educational status, place of residence, main water source and toilet facility, gravidity/parity, ANC visits, nausea/vomiting, changing food intake, meal frequency and pattern, intake of iron/folate or de-worming tablets, MUAC and gestational age). Crude odds ratio (COR) with 95% confidence intervals (CIs) was also calculated for each independent factor.

Multivariable conditional logistic regression was used to identify the independent predictors of anaemia by calculating the adjusted odds ratio (AOR) with 95% CI. The predictors that had a *p* < 0.25 in the bivariate logistic regression model were entered into a multivariable logistic regression model. Multivariable logistic regression can help to adjust for confounders, and *p* < 0.05 was taken as significant.

## Results

### Socio-demographic, maternal and family characteristics

A total of 417 pregnant women participated in the study (*n* = 105 [25%] cases; *n* = 312 [75%] controls). The mean (±*SD*) age of cases was 26.2 (±4.5) years and of controls was 26.3 (±5.0) years. Around 58% of cases and 55% of controls were aged 25–34 years, respectively. The proportion of rural participants in both cases and controls was similar: 42% in cases and 44% in controls. Nearly two-fifths of the cases (41%) and 21% of controls did not attend formal education. The majority of cases (57%) and controls (51%) were pregnant for the first time. A large proportion of cases (71%) and controls (70%) had experienced nausea/vomiting during pregnancy. Similar proportions of cases (65%) and controls (65%) were in the third trimester of pregnancy. About 39% of women in the case group and 29% of women in the control group had low MUAC measurements (<23 cm). The mean (±*SD*) weight in cases was 58.5 (±6.5) kg and in controls was 58.8 (±7.4) kg (Table 1).

**Table 1.** Sociodemographic, maternal and family characteristics of study participants, North Shewa, 2019.

Variables	Cases *n* (%)	Controls *n* (%)	Total *n* (%)
Age (years), *M* (*SD*, IQR)	26.2 (4.5, 22–29)	26.3 (5.0, 23–29)	26.3 (4.9, 22–29)
Age group (years)			
15–24	37 (35.2)	115 (36.9)	152 (36.5)
25–34	61 (58.1)	170 (54.5)	231 (55.4)
≥35	7 (6.7)	27 (8.7)	34 (8.2)
Place of residence			
Urban	61 (58.0)	175 (56.0)	236 (56.6)
Rural	44 (42.0)	137 (44.0)	181 (43.4)
Religion			
Orthodox	97 (92.4)	300 (96.0)	397 (95.2)
Other	8 (7.6)	12 (3.9)	20 (4.8)
Ethnicity			
Amhara	98 (93.3)	306 (98.1)	404 (96.9)
Other	7 (9.7)	6 (1.9)	13 (3.1)
Educational status			
No education	43 (41.0)	66 (21.2)	109 (26.1)
Primary	23 (22.0)	120 (38.4)	143 (34.3)
Secondary	24 (23.0)	81 (26.0)	105 (25.2)
Tertiary	15 (14)	45 (14.4)	60 (14.4)
Marital status			
Single	6 (5.7)	13 (4.2)	19 (4.6)
Married	97 (92.4)	290 (93.0)	387 (92.8)
Divorced/widowed	2 (1.9)	9 (2.9)	11 (2.7)
Occupation			
Farmer	21 (20.0)	68 (21.8)	89 (21.3)
Merchant	23 (21.9)	66 (21.2)	89 (21.3)
Government employee	21 (20.0)	46 (14.7)	67 (16.1)
Housewife	29 (27.6)	103 (33.0)	132 (31.7)
Other^a^	11 (10.5)	29 (9.3)	40 (9.6)
Drinking water source^b^			
Improved	86 (81.9)	272 (87.2)	358 (85.9)
Unimproved	19 (18.1)	40 (12.8)	59 (14.1)
Latrine facility^c^			
Improved	60 (57.1)	201 (64.42)	261 (62.6)
Unimproved	45 (42.9)	111 (35.6)	156 (37.4)
Monthly income (ETB), *M* (*SD*)	3558.6 (2466)	2916.06 (1891.8)	3078.0 (2065)
First pregnancy			
Yes	60 (57.1)	160 (51.3)	220 (52.8)
No	45 (42.9)	152 (48.7)	197 (47.2)
Previous antenatal care visits			
Yes	38 (36.2)	136 (43.6)	174 (41.7)
No	67 (63.8)	176 (56.4)	243 (58.3)
Nausea/vomiting			
Yes	75 (71.4)	220 (70.5)	295 (70.7)
No	30 (28.6)	92 (29.5)	122 (29.3)
Alcohol consumed during pregnancy			
Yes	17 (16.2)	40 (12.8)	57 (13.7)
No	88 (83.8)	272 (87.2)	360 (86.3)
Trimester			
First	2 (1.9)	19 (6.1)	21 (5.0)
Second	35 (33.3)	89 (28.5)	124 (29.7)
Third	68 (64.8)	204 (65.4)	272 (65.2)
Weight, *M* (*SD*)	58.5 (6.5)	58.8 (7.4)	58.8 (7.2)
Height (cm), *M* (*SD*)	159.7 (6.5)	159.9 (6.3)	159.9 (6.3)
MUAC (cm), *M* (*SD*)	23.37 (2.3)	23.8 (2.2)	23.7 (2.3)
<23	41 (39.1)	90 (28.9)	131 (31.4)
≥23	64 (61.0)	222 (71.2)	286 (68.6)

*Note.* IQR: interquartile range; MUAC: mid-upper arm circumference; SBP: systolic blood pressure; DBP: diastolic blood pressure; ETB: Ethiopian birr; ^a^ daily labourer, private employee or self-employed; ^b^ water source = improved (public tap, pipe water at home or protected spring) and unimproved (well water, unprotected spring or river water); ^c^ Latrine facility = Improved (pit latrine with slab, ventilated improved pit latrine, flush or pour flush toilet) and Unimproved (no latrine facility/bush/field or pit latrine without slab/open pit).

### Maternal feeding habits/practices

Both cases and controls had similar feeding practices before pregnancy; 72% of cases and controls ate three times a day before pregnancy. On becoming pregnant, 35% of cases and 49% of controls did not change their food intake. Around one-fifth (21%) of cases and a quarter (26%) of controls reported avoiding certain foods once they became pregnant. A further 33% of cases and 10% of controls reported reducing their food intake (size and/or frequency) once they were pregnant. The majority of cases (45%) and controls (40%) consumed two main meals and two snacks daily during pregnancy, with the majority of participants having a low DDS (53% of cases and 40% of controls) (Table 2).

**Table 2.** Maternal feeding habits/practices of study participants, North Shewa, 2019.

Variables	Cases *n* (%)	Controls *n* (%)	Total *n* (%)
Eating patterns before pregnancy			
Once/twice	2 (1.9)	16 (5.1)	18 (4.3)
Three times	76 (72.4)	226 (72.4)	302 (72.4)
Four or more times	27 (25.7)	70 (22.4)	97 (23.3)
Changed food intake this pregnancy			
No change	37 (35.2)	154 (49.4)	191 (45.8)
Increased size and number	33 (31.4)	128 (41.0)	161 (38.6)
Decreased size and number	35 (33.3)	30 (9.6)	65 (15.6)
Changed snack intake this pregnancy			
No change	56 (53.3)	173 (55.5)	229 (54.9)
Increased size and number	35 (33.3)	114 (36.5)	149 (35.7)
Decreased size and number	14 (13.3)	25 (8.01)	39 (9.35)
Avoidance of food this pregnancy			
Yes	22 (21.0)	82 (26.3)	104 (24.9)
No	83 (79.1)	230 (73.7)	313 (75.1)
Additional foods consumed this pregnancy			
Yes	53 (50.5)	142 (45.5)	195 (46.8)
No	52 (49.5)	170 (54.5)	222 (53.2)
Typical meal pattern this pregnancy			
2 main and 1 small	45 (42.9)	141 (45.2)	186 (44.6)
2 main and 2 main	47 (44.8)	124 (39.7)	171 (41.0)
2 main and 3+ small	13 (12.3)	47 (15.1)	60 (14.4)
Fasting during this pregnancy			
Yes	35 (33.3)	80 (25.7)	115 (27.6)
No	70 (66.7)	231 (74.3)	301 (72.4)
Craving food this pregnancy			
Yes	46 (43.8)	128 (41.0)	174 (41.7)
No	59 (56.2)	184 (59.0)	243 (58.3)
Taking supplements this pregnancy^a^			
Yes	37 (35.2)	121 (38.8)	158 (37.9)
No	68 (64.8)	191 (61.2)	259 (62.1)
De-worming			
Yes	26 (25.2)	88 (28.2)	114 (27.5)
No	77 (74.8)	224 (71.8)	301 (72.5)
Dietary diversity score			
≥5 (good)	49 (46.7)	187 (59.9)	237 (56.8)
<5	56 (53.3)	125 (40.1)	180 (43.2)

^a^ Iron/folate or multivitamins.

## Propensity score analysis

The propensity score model was assessed using the *c* statistic and Hosmer–Lemeshow statistics. The Hosmer–Lemeshow goodness-of-fit test (*p* = 0.177) and the *c* statistic (0.72) indicated good calibration and discrimination ability of the propensity score model.

**Figure 1.**
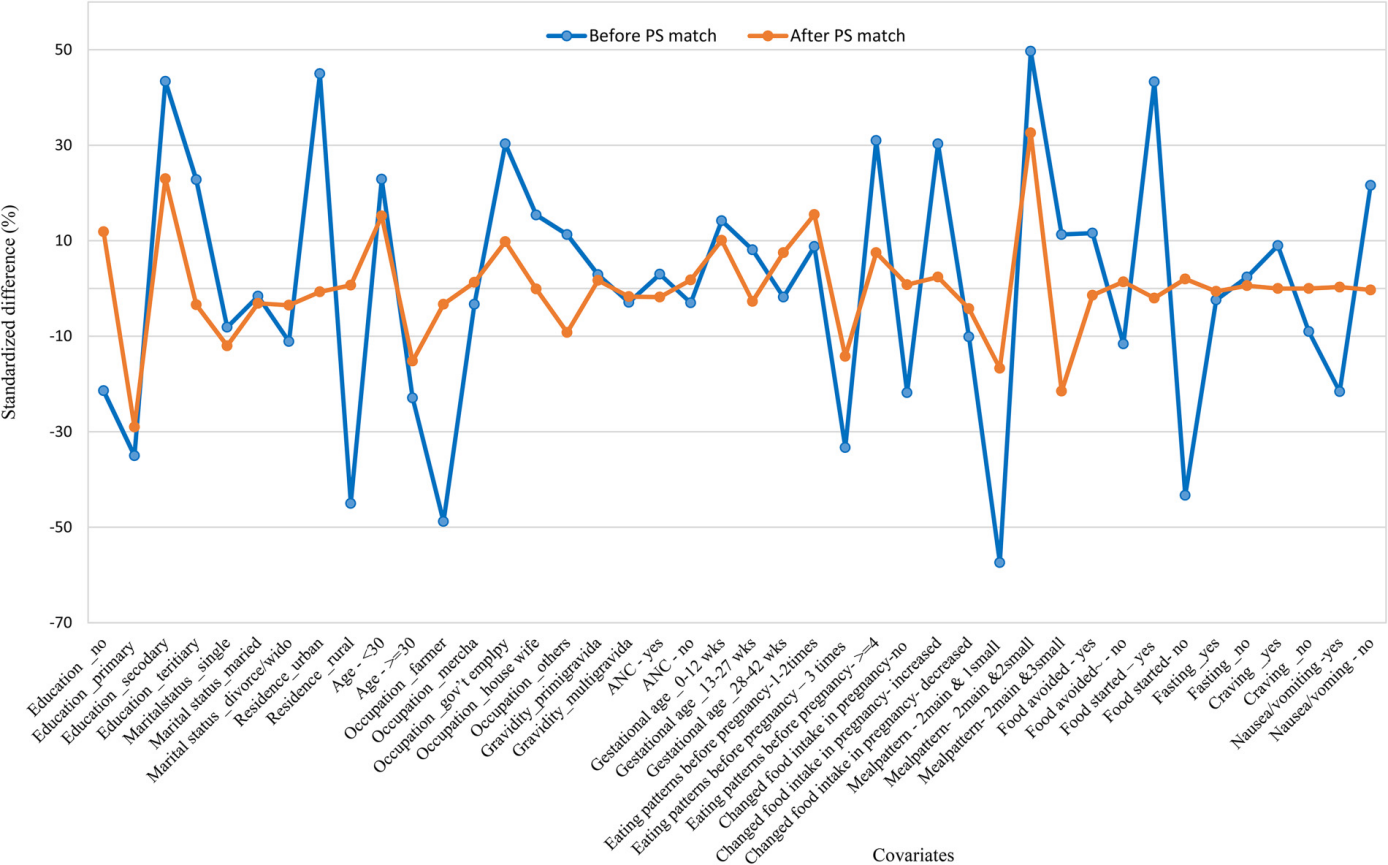
Standardised mean difference before and after propensity score matching comparing covariate values for study subjects having high dietary diversity score and low dietary diversity score.

The covariate balance check indicated that except educational status and meal patterns, all of the variables had less than 0.25 absolute mean standardised difference, which is in the range of the recommended cut-off values (<0.25) (Rubin, 2001) as presented in Figure 1.

### The factors for the occurrence of maternal anaemia among pregnant women

#### Bivariate analysis

The association between predictor variables and maternal anaemia was assessed using a conditional logistic regression model. The bivariate analysis indicated that low educational status, a MUAC < 23 cm, decreasing size and frequency of food intake after becoming pregnant and low DDS were significantly associated with higher odds of anaemia among pregnant women. The odds of anaemia in women with no education were 2.53 times higher than women with tertiary education (COR = 2.53; 95% CI: 1.07, 5.95). Similarly, those who had decreased the size and frequency of food intake after becoming pregnant were at higher odds of anaemia compared to participants who had increased the size and frequency of food intake after becoming pregnant (COR = 4.51; 95% CI: 2.39, 8.89). However, there was no significant difference concerning MUAC, gestational age, water source, latrine facility, alcohol consumption or ANC visits and odds of anaemia (Table 3).

**Table 3.** Determinant factors of anaemia among pregnant women in the selected public health facilities of North Shewa zone, Ethiopia: conditional logistic regression analysis, 2019.

Variables	COR	95% CI	*p*	AOR (95% CI)
Age (years)				
15–24	1			
25–34	1.08	0.68, 1.74	0.74	
≥35	0.75	0.30, 1.88	0.55	
Place of residence				
Urban	1			
Rural	0.77	0.44, 1.37	0.38	
Educational status				
No education	2.53	1.07, 5.95	0.03	3.13 (1.18, 8.32)
Primary	0.69	0.31, 1.53	0.37	0.55 (0.23, 1.29)
Secondary	0.99	0.47, 2.12	0.99	0.77 (0.34, 1.78)
Tertiary	1			1
Occupation				
Farmer	0.42	0.16, 1.11	0.08	
Merchant	0.64	0.30, 1.38	0.03	
Housewife	0.56	0.28, 1.12	0.10	
Other*	0.85	0.35, 2.05	0.72	
Government employee	1			
Water source				
Unimproved	1.53	0.78, 3.02	0.22	0.97 (0.42, 2.25)
Improved	1			1
Latrine facility				
Unimproved	1.37	0.84, 2.25	0.21	1.37 (0.72, 2.62)
Improved	1			1
Parity				
Primigravida	1.29	0.83, 2.02	0.25	1.29 (0.77, 2.18)
Multigravida	1			1
Antenatal care visit so far				
Yes	0.74	0.47, 1.16	0.19	0.75 (0.43, 1.31)
No	1			1
Experienced nausea/vomiting				
Yes	0.98	0.59, 1.63	0.93	
No	1			
Alcohol consumed during pregnancy				
Yes	1.33	0.72, 2.47	0.37	
No	1			
Gestational age				
First	1	1		1
Second	3.37	0.75, 15.25	0.11	3.91 (0.82, 18.68)
Third	2.84	0.64, 12.58	0.17	4.12 (0.87, 19.46)
MUAC				
<23 cm	1.53	0.95, 2.47	0.08	1.33 (0.75, 2.38)
≥23 cm	1	1		1
Changed food intake in this pregnancy				
No change	0.97	0.56, 1.73	0.96	0.79 (0.42, 1.46)
Decreased size and number	4.51	2.39, 8.89	0.01	6.89 (3.23, 14.70)
Increased size and number	1	1		1
Typical meal pattern				
2 main and 1 small	1.02	0.43, 2.41	0.97	
2 main and 2 main	1.37	0.64, 2.91	0.42	
2 main and 3+ small	1	1		
Fasting during pregnancy				
Yes	1.52	0.93, 2.47	0.09	1.52 (0.87, 2.65)
No	1			1
Craving food				
Yes	1.14	0.73, 1.79	0.56	
No	1			
Taking supplements				
Yes	0.86	0.54, 1.36	0.51	
No	1		1	
De-worming				
Yes	0.85	0.51, 1.42	0.55	
No	1			
Dietary diversity score				
<5	1.82	1.13, 2.94	0.014	2.14 (1.24, 3.69)
≥5 (good)	1			1

*Note.* COR: crude odds ratio; AOR: adjusted odds ratio; MUAC: mid-upper arm circumference; *E.g., daily labourer, private employee or self-employed.

#### Multivariable analysis

The multivariable logistic regression analysis showed that a low DDS (AOR = 2.14; 95% CI: 1.24, 3.69), reducing food intake (AOR = 6.89; 95% CI: 3.23, 14.70) and having no formal education (AOR = 3.13; 95% CI: 1.18, 8.32) were associated with an increased odd of anaemia. Similarly, some variables, such access to an unimproved latrine facility (AOR = 1.37; 95% CI: 0.72, 2.62), primigravida (AOR = 1.29; 95% CI: 0.77, 2.18), a MUAC < 23 cm (AOR = 1.33; 95% CI: 0.75, 2.38), fasting (AOR = 1.52; 95% CI: 0.87, 2.65) and previous ANC visit (AOR = 0.75; 95% CI: 0.43, 1.31), were positively associated with an increased odds of anaemia, though these were not statistically significant (Table 3).

## Discussion

The findings of this study indicate that a low DDS, reducing food intake and having no formal education were significant predictors of anaemia among pregnant women in North Shewa, Ethiopia. This study revealed that women who had low DDS were two times more likely to have anaemia compared to women with a higher DDS. This finding is in line with previous studies conducted in Eastern Ethiopia (Abriha et al., 2014, Kedir et al., 2013), which have reported higher odds of anaemia in women with a low DDS. Similarly, the results of studies conducted in northwest Ethiopia (Alem et al., 2013), Southern Ethiopia (Lebso et al., 2017, Gebremedhin et al., 2014) and Ghana (Saaka and Rauf, 2015) have also indicated that a low DDS is associated with an increased odds of anaemia. Conflicting results have been reported in studies from Pakistan (Ali et al., 2014) and Ghana (Saaka et al., 2017), possibly due to measurement differences. Our results showed that women who reduced their dietary intake were more likely to be anaemic compared to those who did not change their dietary intake. Similar results have been reported in studies conducted in Ethiopia (Kedir et al., 2013) and Mali (Ayoya et al., 2006).

The association of a high DDS with the low occurrence of anaemia might be due to sufficient and diversified food intake during pregnancy, which helps to meet required nutrient recommendations (including for iron) and prevents unintended consequences such as anaemia (Lartey, 2008). Furthermore, this might also be because an increase in a woman's DDS is a good proxy indicator of micronutrient adequacy in her diet (Arimond et al., 2010) as well as a positive indicator of nutritional status (Zhang et al., 2017). Reducing dietary intake during pregnancy might lead to insufficient food intake, which could result in low iron intake (Bhargava et al., 2001) and a subsequent lack of macro- and micronutrients. Thus, it is important to provide dietary counselling to women during pregnancy.

In agreement with other studies conducted in Ethiopia (Gebre and Mulugeta, 2015), the present study showed that having no formal education was associated with higher odds of anaemia among pregnant women compared with those who had completed tertiary education. However, the findings of previous studies did not demonstrate an association between maternal education and the risk of anaemia (Alemu and Umeta, 2015, Asres et al., 2014, Kedir et al., 2013, Kefiyalew et al., 2014, Melku et al., 2014). The discrepancy in study results might be due to the differences in population and methodology, as the present study was a case–control study, whereas previous studies were cross-sectional.

Furthermore, living in a rural area was associated with a higher occurrence of anaemia during pregnancy (Gebre and Mulugeta, 2015, Kefiyalew et al., 2014). However, other studies have reported that the odds of anaemia were not significantly different between rural and urban residents (Alemu and Umeta, 2015), which is in line with our study results. The gravidity of women was identified as a predictor in several studies conducted in Ethiopia (Abriha et al., 2014, Asres et al., 2014, Gebremedhin et al., 2014, Lebso et al., 2017, Kedir et al., 2013). Our study and a cross-sectional study from Ethiopia have indicated that gravidity was not associated with increased odds of anaemia (Kefiyalew et al., 2014). This might be due to methodological differences, as the present study was a case–control study, but the previous studies were cross-sectional.

Different observational studies report that iron/folate supplementation was associated with lower odds of anaemia during pregnancy (Gebre and Mulugeta, 2015, Lebso et al., 2017). In contrast, the present study and another study conducted in northwest Ethiopia (Alem et al., 2013) have indicated that the odds of anaemia were not significantly different between those who had supplemented with iron/folate and those who did not. Similarly, evidence has shown that intermittent iron supplementation (Pena-Rosas et al., 2015), supplementation with folic acid only or with other micronutrients (Lassi et al., 2013) and the provision of multiple micronutrients plus an iron and folic acid supplement (Haider and Bhutta, 2015) during gestation failed to improve maternal anaemia. A possible explanation could be different side effects. Women might not adhere to supplementation guidelines, which may dilute the effect of iron supplementation on anaemia towards the null.

The strength of this study is the use of the case–control study design, which is an ideal design and important for identifying potential factors. Another strength of this study is the application of an advanced statistical analysis (propensity score analysis), which can help to reduce selection and confounder bias. However, the possible limitations of this study are social desirability and recall biases, as this study relied on self-reporting and the memories of participants. Further, a case–control study can only identify an association; it does not establish a temporal or cause–effect relationship. Due to resource constraints, it was impossible to measure and incorporate all potential predictors of maternal anaemia. As a result, there could be an under- or over-inflation of reported effect sizes, and the interpretation of the study's results should consider these limitations.

## Conclusion

This study demonstrated that intake of a less diversified diet, reducing food intake and having no formal education were associated with higher odds of anaemia. Interventions focused on dietary counselling should be considered to strengthen existing prenatal health service programs. Women who are pregnant or considering pregnancy should be strongly encouraged to increase, rather than decrease, both the diversity and amount of food intake during pregnancy to ensure they meet macro- and micronutrient requirements.

## Supplemental Material

sj-doc-1-nah-10.1177_02601060231152345 - Supplemental material for The effect of dietary patterns on maternal anaemia in North Shewa, Ethiopia: A case–control study with Propensity Score Analysis Supplemental material, sj-doc-1-nah-10.1177_02601060231152345 for The effect of dietary patterns on maternal anaemia in North Shewa, Ethiopia: A case–control study with Propensity Score Analysis by Kelemu Tilahun Kibret, Catherine Chojenta, Ellie D’Arcy and Deborah Loxton in Nutrition and Health
